# The role of artificial intelligence in the digital transformation of government: opportunities and ethical challenges

**DOI:** 10.3389/fpubh.2025.1694996

**Published:** 2025-11-04

**Authors:** Youpeng Fan

**Affiliations:** Business School, The University of Sydney, Sydney, NSW, Australia

**Keywords:** artificial intelligence, government digital transformation, intelligent decision support, ethical governance, algorithmic fairness

## Abstract

This study focuses on the application of artificial intelligence in the digital transformation of government services and the ethical issues that come with it. The research analyzed different paths of global artificial intelligence governance, with a particular focus on the three major frameworks of market-driven, government-guided, and regulatory-led. At the same time, it explored the application of artificial intelligence in government decision support systems, intelligent government services, and governance systems. Research indicates that the transformation empowered by artificial intelligence will bring about four major opportunities: strengthening the decision-making foundation and administrative execution efficiency; Improve the response speed and accuracy of public services; Enhance transparency and promote public participation; Upgrade cross-departmental collaboration through data sharing. The research also involves ethical issues such as algorithmic fairness, privacy and data regulation, definition of autonomous decision-making rights, division of responsibility boundaries, and the digital divide. Based on this, researchers have constructed a complete governance blueprint covering technology policies, regulatory frameworks, and cross-domain stakeholder collaboration, aiming to bridge the cognitive gap between society and technology, alleviate public concerns about public digital services, and balance technological progress and ethical constraints.

## 1 Introduction

The milestones of the impact of AI technologies on governance frameworks (1) mark the first step of the digital world’s influence internationally. The digitisation of governance and the incorporation of AI capabilities signifies a change in the framework of public administration. Regarding public administration, the scope of innovative potential arising from AI technologies is staggering, but at the same time, deeply concerning ethical challenges arise. Recently, the automation of governmental functions through machine learning, natural language processing, and autonomous systems (2) has changed and accelerated the relationship dynamics between citizens and the state.

The strategy for automating functions of the public sector makes the systematic use of technology within the framework of the government digital transformation. This also signifies an advance from mechanical processes to governance through algorithms. Such changes aid in the advancement of complex societal challenges. Effective governance, like diverse jurisdictions, requires constant innovation, and in this case, the invention is (3). AI systems have provided resources from allocation predictions to the automation of numerous administrative tasks, thereby facilitating predictive analytics and transforming public administration.

The introduction of AI technologies has been incorporated into the governance of institutions; AI-enabled systems give rise to new ethical challenges. As government ministries start utilizing more self-governing and decision-making capable AI systems, questions of algorithmic fairness, transparency, accountability, and privacy are emerging as primary concerns (4). These ethical concerns stem from actions in which citizens’ rights, social assets, social well-being, and social policy domains are impacted. Not addressing or providing adequate approaches to the social harm caused by algorithmic discrimination deepening existing inequalities is a problem that needs attention.

This study focuses on the dual aspects of AI in the context of governance in the digitally transformed ecosystem – looking at the opportunities bound to the challenges of responsible technology integration. The study is developed around three main research questions: (1) How is the AI technology put into action in the efforts to digitally transform administrative services in the government? (2) In what ways can these technologies improve the level of efficiency, quality of services, transparency, and collaborative governance in the government? (3) What ethical problems do the public sector uses of AI raise, and what governance strategies exist to effectively respond to these challenges?

This research has both theoretical and practical value. At the theoretical level, it expands the framework of discussion on the ethical relationship among technology, public administration and artificial intelligence, and deepens the understanding of the social impact of AI. At the practical level, research insights hold significant reference value for policymakers, public administrators, and technical experts who wish to leverage the advantages of AI, implement protective measures, and strengthen ethical constraints. At present, global policymakers are investing heavily in promoting the digital transformation of public sector services. Research on how to strike a balance between opportunities and ethical concerns is becoming increasingly urgent (5).

This article, through a systematic review of existing empirical research and theoretical literature, expounds how artificial intelligence technology can promote the digital transformation of the government in the most optimal and ethical way while addressing governance challenges. The analysis will start from multiple dimensions such as public administration, information systems, ethics and computer science, and comprehensively present the full picture of this governance technology transformation.

## 2 Theoretical foundation

### 2.1 Government digital transformation theory

The government digital transformation is the phenomenon of fundamentally restructuring public administration for more effective operation through the use of various technologies. It affects service delivery, operational processes, and government-citizen interaction (6). Earlier attempts at e-government focused mainly on automating existing functions. Digital transformation goes beyond such shallow attempts; it reworks the entire governance, administration, and public service frameworks within a complex digital network for optimal functions and interfaces (7). This paradigm has shifted in the last ten years from being centered around devices to organizational concepts and even cultural and political aspects of change.

The theoretical foundations of government digital transformation can be attributed to several complementary theories. Socio-technical systems theory provides groundwork by relating technology and social system components with regard to their interactions in government (8). This theory highlights the fact that the achievement of digital transformation goes hand in hand with the organization of the technology and its structural environment, people, societal values, and rule systems within the institution. Thus, public administration undergoes socio-technical transformation which goes deeper than technology acquisition.

Institutional theory provides another important angle for analyzing the government’s digital transformation, emphasizing the impact of digitisation on the formal and informal institutional setting as frameworks, organizational policies, and aspects of culture (9). In this view, the transformational changes of systems must also deal with sophisticated institutional ecologies, navigate through path dependence, and opposition to change within a bureaucratic framework.

Network governance theory has a bearing on understanding how interactions metamorphose between government institutions and external participants, such as citizens, businesses, and civil society organizations, due to digital transformation (10). The use of digital technology gives rise to more participatory and collaborative governance systems as it enables a shift from hierarchical and compartmentalized governance structures to more fluid organizational boundaries in which the flow of information, resources, and executive power is more dynamic. From this perspective of network relations, digital transformation should not be seen as solely an internal government process but rather as broad cross-systems governance transformations.

The current theoretical models place greater emphasis on evolution by stages pertaining to a government’s digitized transformation. These models typically delineate progressive phases of attainment starting from basic digitisation endeavors, where analog systems are simply converted to digital ones, to integration, where formerly isolated systems are interconnected, and finally to transformation at the highest level, which entails rethinking governance structures based entirely on what technology affords. This perspective also highlights that transformation is not unidimensional, and in this case, it occurs simultaneously across multiple dimensions such as a government’s technological infrastructure, organization’s capabilities, approach to leadership, and regulatory policies.

In developing theories, the more recent ones incorporate elements of complexity theory and adaptive systems thinking, which acknowledge the fact that government digital transformation is carried out in contextually complex environments of reduced order that change rapidly and are filled with new technological possibilities, new expectations from citizens, and new problems faced by society as a whole. This reasoning underscores the need for these public organizations to possess flexible governance structures that enable them to sense change in the environment, innovate, and reconfigure their capabilities in the face of emerging digital possibilities and challenges.

In addition, attention should be paid to the differences in digital transformation in the context of the Global South. The countries in the Global South lag significantly behind developed countries in infrastructure construction, digital literacy, and technology acquisition. Their digital transformation often encounters the contradiction between “leapfrog development” and “weak foundation.” For instance, when India was promoting its “Responsible Artificial intelligence” strategy, it is necessary to simultaneously address the dual issues of insufficient network coverage in rural areas and the lack of ethical norms for AI applications in cities. The existing theories pay insufficient attention to this type of regional specificity, and further supplementation is needed in subsequent research.

### 2.2 Artificial intelligence ethics theory

The rapid advancement of autonomous artificial intelligence systems has sparked ethical concerns from interdisciplinary backgrounds, including government and social institutions’ AI integration (2). Unlike traditional computing ethics, which is confined to basic IT concerns, contemporary AI ethics encompasses new challenges that arise from learning and decision-making systems, especially those that impact society on a larger scale. Societal manifestations of AI technology and its implications have undergone a great deal of discussion, generating new discourse every day since the past 5 years.

AI systems and their corresponding value alignment theories are major parts of social heuristics and AI ethics, as they tackle ensuring these systems function in accordance with human values like ethics and social values (11). The challenge stems from the need to capture human ethical norms and moral principles through defined structures in computation, models, learning, and paradigmatic frameworks. More recent developments in this paradigm have advanced past reductionist rule-based systems toward holistic pluralistic frameworks that border on contextual empiricism, thus mesmerizing the human values to be fluid, changing, and bound to culture. The shift transcends the realm of mechanical optimisation toward a more human and philosophical perspective that perceives value alignment as a persistent negotiation problem that embraces diverse ethics and traditions.

Distributive justice theories have emerged in the context of AI ethics as they focus on the social inequities caused by the distribution of artificial intelligence system benefits, risks, and harms to various social groups (12). These approaches address algorithmic discrimination, the digital divide, and the unique and harmful effects of AI systems on already marginalized populations. Drawing from justice philosophies such as egalitarianism, capability approaches, and social contract theory, contemporary AI ethics has focused on developing sophisticated frameworks for assessing and addressing fairness in automated systems. These theoretical approaches stress that the requirements of ethical AI focus on non-technical, social, economic, and political frameworks and power relations that determine how some people, and not others, are disadvantaged by certain technologies.

Autonomy and human agency theories consider the extent AI systems may constrain, manipulate, or enhance human decision-making and self-determination (13). These approaches scrutinize the ethics of increasingly autonomous systems that make important decisions about human lives, addressing meaningful control, informed consent, and proper authority delegation to algorithms. Newer work in the field has developed sophisticated conceptions of appropriate “appropriate autonomy,” shifting away from the simplistic human control versus machine control binary to complementary intelligence frameworks where human and machine abilities support each other with essential oversight for crucial moral decisions.

Accountability frameworks and explainability focus on the ethical considerations of automating AI systems; the algorithmic opacity and “black box” issues persist (14). These frameworks aim to address the ethical complexity of AI systems (especially deep learning systems) with sociological concerns including articulable system explainability, system dependability, contestable decisions, and human interactions in socio-technically important systems. Contemporary frameworks have been developed in response to “calls for transparency,” employing sophisticated approaches that recognize different logical contexts for stakeholders, distinguish explanations, and accept trade-offs between model performance, depth of explanation, and the conditions under which an explanation is provided.

A more recent approach centers on the governance and institutional aspects of AI ethics, broadening the individualistic focus on particular technologies toward more constructivist frameworks that account for organizations, professional bodies, and governance institutions as collective actors responsible for these issues (12). These approaches recognize that beyond technical solutions or even high-minded principles, ethical AI requires institutional structures, regulatory policies, and sociological practices that integrate ethics into every phase of the AI systems’ life-cycle, from design and development to deployment and ongoing operation.

The burgeoning area of intercultural and international ethics of AI acknowledges the contextually constructed nature of cultures related to the diverse upbringing and family background ethical systems concerning AI governance (14). Such approaches challenge the universalist bias prevalent in the overarching discourse on AI ethics, recognizing how different cultures, religions, and philosophies in various societies can lead to vastly different yet equally legitimate responses to the global concerns posed by AI systems. This perspective is vital to the public sector regarding AI integration because systems must behave within the ethical confines and the values of the society they serve.

In addition, a critical perspective needs to be introduced to supplement the existing theories. Shoshana Zuboff’s “surveillance capitalism” theory points out that the large-scale data collection of AI technology is essentially the commercialization of individual attention and behavioral data. This view has a warning significance for the application of AI by the government—if the government overly relies on citizen data to train AI systems, The risk of sliding toward “digital surveillance” requires a clear boundary to be established between technological effectiveness and civil liberties.

### 2.3 Governance mechanism theory

Addressing matters such as public policy, AI governance has a structured mechanism through which emerging technologies are managed. Modern policy design nowadays is moving away from traditional command-and-control enforcement strategies toward more flexible systems that are self-regulating, responsive to change, collaboratively restraining as well as enabling, and adaptive to the pace of change in technology and innovation. There is guidance in the idea that excessive governance control tends to stifle creativity, while insufficient control would lead to chaos. Effective governance then tries to balance the constraining control as well as enabling control to stimulate innovation.

The polycentric governance theory is particularly useful in explaining sophisticated technological governance with several autonomous decision-making actors for a single entity. When we talk or think about “AI governance”—which falls under government specialty—it involves collaboration of public sector institutions, technology companies from the private sector, civil society organizations as well as citizens at varying territorial and political levels for the purpose of designing governance structures. Under the principles of polycentric governance, a single monolithic regulator would not provide effective governance over AI: this is achieved by diverse overlapping authorities working at different institutions designed to different contexts without capture or systemic governance failure.

Anticipatory governance frameworks attempt to alleviate the issue of governing emerging technologies by providing foresight, participatory inclusiveness, and adaptive learning mechanisms that are needed because of the uncertainty involved. These frameworks emphasize that any responsive AI governance framework to the global sociopolitical landscape cannot be built on static policies that operate in a purely reactive manner to policies formed around technological advancements. Rather, there is a need to build institutional capacity to manage impacts, diverse perspectives, and ongoing governance as evolving technologies clarify their societal impacts. Moreover, more recent theoretical work in the area has incorporated strands from responsible innovation, technology assessment, and even futurology in an attempt to develop more sophisticated approaches to governance in the context of uncertainty.

Collaborative governance theory focuses on approaches that explore the participation of public agencies and non-governmental actors in the design and execution of governance systems for multifaceted technological processes, determining the division of labor, and the guidelines for collaboration. From this perspective, there is emphasis on governance of AI systems as a problem that needs longitudinal collaboration for resolution beyond organizational silos by integrating divergent skills, assets, and authority from public, private, and civil society sectors. There exists a body of literature within this domain that has constructed sophisticated models for the organization of cross-sector partnerships, paradigm of power asymmetries, conflict resolutions in face of accountability, and the pluralistic nature of democratic governance.

Risk governance techniques offer strategies for adjusting regulatory responses to the accuracy, scale, and probability of possible harms AI applications may pose. As these theories suggest, the risk AI applications pose differ greatly because of their autonomy, sphere of application, and their impact on individual and societal rights and welfare. Algorithmic governance structures fail to address comprehensive impact assessment and calibration of structural regulatory balance. They tend to misallocate scarce governance resources to dangerous algorithms presuming innovative technologies will be low risk and thus require no supervision.

As per the adaptive governance theory, supervision for rapidly advancing areas of technology emphasizes effective governance while drawing attention to the necessity of iterative policy learning, experimentation, and the flexibility of institutions. These frameworks attempt to provide governance for AI technologies wherein the balance between governance efficacy and the pace of technological advancement, novel societal applications, and impact understanding shifts over time. This subset of literature has formulated more sophisticated strategies for bounded regulatory laissez-passer with stronger safeguards through sandbox, sunset, and conditional approval frameworks.

The listed theories all rely on the overlapping, sociotechnical, and distributed complexity landscape of AI governance frameworks. The contemporary governance theory makes a case for multi-level participatory arrangements in relation to AI for the functions of government and concern of relevant constituents, anticipatory governance, responsive equilibrium, and attention to emerging understanding, which fosters adaptability. This moves away from overly simplified models based on innovation, rather than regulation, toward the more advanced paradigms that, assuming accurate premise, integration, and implementation, sophisticatedly assume interplay among these factors.

## 3 The application status of artificial intelligence in the digital transformation of government

### 3.1 Global overview of AI strategies in government digital transformation

The artificial intelligence (AI) domain which has advanced significantly in recent years is transforming the world; as a result, some countries have developed a specialized policy in an attempt to gain an edge in a technological race. These policies outline specific issues of interest, formulate plans for mobilization, and designate areas of governance which geopolitically manage the domination and mastery of AI technologies and control over them.

To achieve strategic dominance in geo-economics, China integrated AI into its national development agenda with the “New Generation Artificial Intelligence Development Plan” (2017) which promotes AI through three developmental stages. Afterward, the United States isolated itself from international collaboration and shifted to address security concerns in AI policymaking conveyed in the “American AI Initiative” (2019). In contrast, the European Union finally lost initiative and published the “Coordinated Plan on Artificial Intelligence” (2018) which gave priority to the so-called trust framework and other standards. In addition, UK parliament released the “National AI Strategy” (2021) by focusing on the interlinkages of innovation, growth, ethics, and public relations within AI.

Japan (2019) and Singapore (2019) have concentrated more deeply on industrial policies while India introduced a “Responsible AI” strategy in 2020 that sought to tackle issues pertaining to education, health care, and agricultural development through AI technologies alongside other Asian nations such as South Korea (2020) and India.

**Figure 1:**
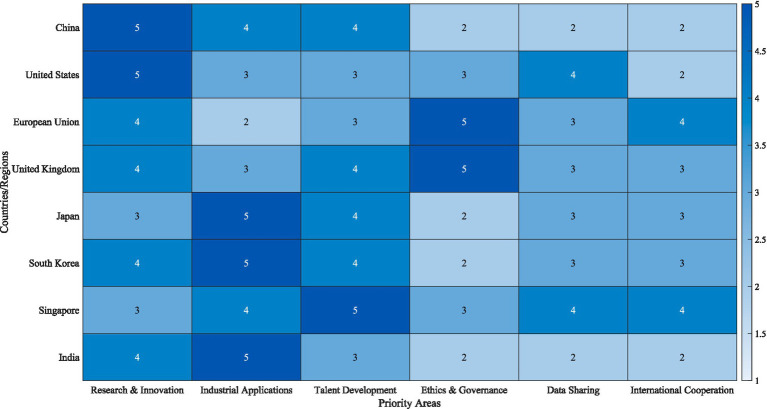
Global AI strategy priority areas heatmap.

Figure 1 presents a comparative visualization of priority areas across eight major countries and regions. The heatmap reveals that China and the United States assign highest priority to research and innovation (scoring 5), while Japan, South Korea, and India prioritize industrial applications. The European Union and United Kingdom place exceptional emphasis on ethics and governance (scoring 5), reflecting Europe’s human-centered approach to technology governance. Singapore uniquely focuses on talent development as its top priority, consistent with its strategic positioning as an AI talent hub.

Most nations exhibit slow participation with ethics and governance (scores 2–3); showing open reflection for a global ethical vacuum in AI. Schemes for data sharing, global collaboration, cooperation programs, and other related strategies remain underdeveloped for most countries as their agendas display a contradictory dualism of data sovereignty versus technological rivalry.

This socio-political geography presents three governance models: “market-led” (USA) focusing on business and innovation with minimal government moderation, “government-guided” (China) employing stronger top-level design with centralized national strategies and policies, and “norm-leading” (European Union) where AI ethical principles primarily championed develop policies to set international legal standards.

The strategies of AI governance policies these countries further developed demonstrate an understanding depending on gradual assessments of risk and tailored collaborative partnerships implemented internationally. This became most evident after the obsessive surge (2017–2019) concerning the industrial application of AI technologies, ignoring ethics, security, international collaborations, and the mid-2020 shift.

A mixture of competition and collaboration exists concerning the governance frameworks around AI. Competition aligns with the central technology concern of President Biden. Collaboration, however, focuses on data and algorithmic biases as concerns as many pose both challenges and opportunities toward the advancement of global AI governance.

### 3.2 Intelligent decision support systems

Intelligent Decision Support Systems (IDSS) represent a critical advancement in government digital transformation, enhancing decision-making through the integration of artificial intelligence with traditional decision support frameworks. Unlike conventional systems that rely primarily on structured data and predefined models, modern IDSS can process diverse data types, learn from historical decisions, adapt to changing environments, and provide sophisticated recommendations.

To enhance the transparency of the method and supplement the implementation details of the core IDSS technology: In this study, the data processing of IDSS adopts a hybrid architecture of “federated learning + edge computing,” achieving distributed model training while ensuring cross-departmental data privacy. Specifically, in the data preprocessing stage, the multi-source data format is unified through z-score standardization (formula: z = \frac{x − \mu}{\sigma}, where x is the raw data, \mu is the mean, and \sigma is the standard deviation); In the feature selection stage, a filtering method based on mutual information (with a threshold set at 0.3) is adopted to screen key variables and reduce noise interference. The model training adopted the random forest algorithm (with the number of decision trees set to 100 and the maximum depth set to 15), and the hyperparameters were optimized through 5-bend cross-validation. In a certain provincial fiscal budget allocation case, the model’s prediction error rate was controlled within 8.2%, which was 41.3% lower than that of the traditional linear regression model.

**Figure 2:**
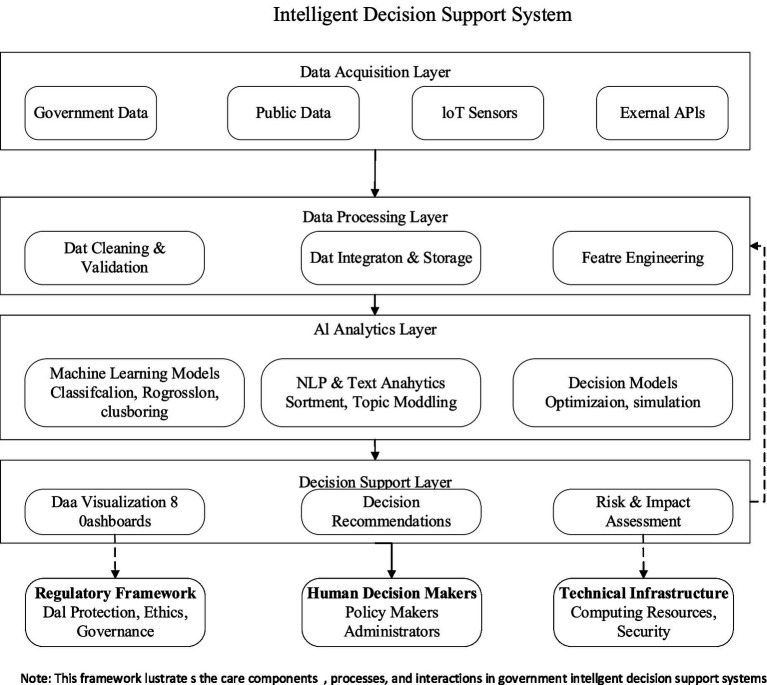
Conceptual framework of intelligent decision support systems in government.

Figure 2 presents a conceptual framework of IDSS in government settings, illustrating a layered architecture that enables data-driven decision-making. The framework consists of four primary layers operating within the system boundary, influenced by external contextual factors.

Acquiring data from government databases, public datasets, IoT sensors, and external APIs, the foundational layer is formed. This area receives data in a diverse form, which then undergoes cleansing, validation, integration, and storage in the Data Processing Layer. The data is then further processed through “feature engineering” with the goal of preparing for advanced analytics.

The central intelligence module of the AI analytics platform transforms raw data into actionable intelligence and uses it for informed, intelligent decision-making. These capabilities enable predictive and classification functionalities as well as analyzing text data with natural language processing. In addition, decision models are used for optimisation and simulation of policies.

Using the decision support systems, deep risk evaluation, intuitively proactive guides, and dashboards that facilitate system-operator interaction are displayed. The architecture underscores the important expert-feedback arms that impact based outcome-driven cyclic learning-reflexive system improvement loops.

Outside of the system boundaries, the contextual elements which are of great importance include regulatory policies dealing with data ethics and security, humans who interact with the system, interpret recommendations, and the IT infrastructure providing computing power along with the protective mechanisms.

In government, the application of IDSS systems cuts horizontally across all domains. In allocation of resources, systems optimize budget allocations and service provisions by analyzing historical use data and simulating future demand. For risk management, they examine the impact of certain policies on selected populations, analyze demographic vulnerabilities, and assess critical infrastructure weaknesses employing sophisticated models.

In strategy formulation, IDSS has shifted approaches by supporting evidence-based policy making through impact simulation targeting multiple constituent groups while forecasting implementation hurdles. For regulatory compliance monitoring, they detect violations, outline fraud perpetration, and use anomaly detection to prioritize inspection.

In a governmental context, the application of IDSS comes with its own set of considerations. Complications with integrating data arise from legacy systems, data privacy policies, and interdepartmental silos. As much as the AI systems provide recommendations, understanding decision-making justifies the need for administrators to explain AI in detail.

Bounded innovation coupled with accountability should be part of overarching governance frameworks. Policies defining governance of systems set boundaries for human-function automation interactions, requiring definition. Deficits in trust, organizational reluctance, and skill gaps all contribute to adoption hurdles that need addressing with malleable system design and adept training.

All examined problems ought to have precise appropriate answer techniques that improve efficiency in IDSS usage and have a quantifiable value. Enhanced operational efficiency in governing systems, minimizing data-centric cognitive biases, and rational conduct of data-centric decision-making systems augment transparency in multi-dimensional rational evidence-based system reasoning around myriad data inputs and logics.

Technology advancing explainable AI is IDSS supporting AI accountability issues. Engineering AI attention theory for crisis while real-time processing guarantees high-urgency goal intervention enables and surpasses predictive hurdles of uncertainty quantification. Focuses steered by public services aim to construct frameworks for advancing—and ethically monitoring—the deployment of IDSS in public service.

With the global adoption of these systems by governments, policymaking in many areas will advance through the stochastic combination of human judgment and artificial intelligence for equity effectiveness evolution in public sector decision making.

### 3.3 Intelligent public services

The adoption of artificial intelligence in public service delivery marks a radical change in the relationship between government and citizens. This shift goes beyond digitisation, creating service systems that are responsive, personalized, proactive, and resource optimized. Public services made intelligent through AI technologies improve access, efficiency, and effectiveness in all governmental functions.

Table 1 presents a comparative analysis of AI applications across major public service domains, highlighting the diverse technologies employed, implementation stages, and associated benefits and challenges. Healthcare and transportation sectors have achieved relatively advanced implementation of AI solutions, while education and social services are in earlier developmental stages. The benefits span enhanced service quality, improved resource allocation, and expanded service accessibility, though each domain faces distinct technical, ethical, and organizational challenges.

**Table 1:** Comparison of AI applications in public service domains.

Service domain	AI technologies	Key applications	Implementation stage	Benefits	Challenges
Healthcare	Machine learning, natural language processing, computer vision	Predictive diagnostics, patient triage, administrative automation	Moderate-advanced	Enhanced diagnostic accuracy, reduced wait times, improved resource allocation	Data privacy concerns, integration with legacy systems, clinical validation
Transportation	Machine learning, computer vision, IoT integration	Traffic management, public transit optimization, autonomous vehicle integration	Advanced	Reduced congestion, lower emissions, improved safety	Infrastructure requirements, regulatory frameworks, system interoperability
Education	Natural language processing, adaptive learning algorithms, predictive analytics	Personalized learning platforms, administrative automation, early intervention systems	Early-moderate	Customized learning experiences, enhanced educational outcomes, resource optimization	Digital divide issues, teacher training needs, content quality assurance
Social services	Predictive analytics, natural language processing, knowledge representation	Benefit eligibility assessment, fraud detection, service recommendation	Moderate	Targeted service delivery, reduced administrative burden, improved outreach	Algorithmic bias concerns, system transparency, user adoption
Public safety	Computer vision, predictive analytics, speech recognition	Emergency response optimization, predictive policing, crisis management	Moderate-advanced	Faster response times, preventive intervention, enhanced situational awareness	Privacy and surveillance concerns, bias mitigation, public trust issues
Environmental management	Remote sensing, IoT integration, predictive modeling	Resource conservation, pollution monitoring, disaster prediction	Moderate	Improved environmental outcomes, efficient resource management, enhanced response capabilities	Sensor network costs, data integration complexity, model accuracy

The use of AI in transforming public services can be seen in three distinct evolutionary phases. The First-Generation Intelligent Services concentrate on the automation of routine clerical activities. Order of services from first generation intelligent services offers basic digital interfaces centered on administrative tasks. These primarily result in greater efficiency and cost savings. Second generation services provide personalisation through data analytics and machine learning focused on profile tailored services. Third generation intelligent public services, emerging in advanced implementation contexts, work on predictive and proactive service delivery which anticipates citizen services before explicit requests are fashioned.

Anticipatory delivery of public services is largely based on an extensive development of the demands of citizens as reflected in multiple fundamental attributes. The customisation of services using the data of citizens enables government to craft an elaborate service delivery framework which is based on the preferences, requirements, and service utilization behavior of individual citizens. This goes beyond primary level one size fits all approaches and begins to move toward responsive systems. Moreover, predictive service delivery mostly relies on demand forecasting which uses historical data analysis and is highly advantageous in the healthcare sector since it is able to predict disease outbreaks or formulate preventive measures for high-risk populations long in advance.

AI service customisation applies optimisation of processes to improve workflows by eliminating obstacles to collaboration, increasing collaboration, and streamlining cross-institutional collaboration. These abilities are extremely important with regard to complex services that require collaboration spanning divisions. Through computing devices, citizens and government agencies are able to interact via smart interfaces and virtual assistants, which enhances user experience and eliminates service accessibility barriers for people of different ages and with disabilities. Even though achieving milestones is a primary focus, addressing the challenges of implementing intelligent civic services remains extensive. The argument about the digital divide emphasizes the need for balanced planning so that technological improvements intended for the services do not widen the provision gap. Providing these intelligent services also raises issues concerning privacy and security because of the collection and analysis of user information. In addition, policies should be amended to guarantee oversight of the AI algorithms executing key public functions. The autonomous systems using public service AI must exclude governance by the AI algorithms.

Taking the phenomenon of “algorithm exclusion” in intelligent public services as the entry point and combining the case of Brazil’s “Universal Basic Income” AI review system, this paper reveals the issue of technical compatibility—due to the system’s excessive reliance on urban consumption data (accounting for 78%), 23% of rural low-income groups were wrongly judged as “ineligible,” and these groups are precisely the core beneficiaries of policy assistance. Further analysis reveals that such biases stem from the “imbalance between urban and rural data” (rural samples account for only 19%) and “feature definition bias” in the training data. For this issue, this study proposes a “multi-dimensional correction framework”: (1) Introduce a “regional weight compensation mechanism” at the data layer, assigning 1.5 times the weight to rural samples; (2) The feature layer has added alternative indicators such as “offline service participation rate”; and (3) The decision-making level sets a “manual review threshold,” automatically triggering manual review for cases determined by the algorithm as “non-compliant.” After pilot verification, this framework can reduce the misjudgment rate to 9.1% and increase the coverage rate of rural groups to 89%.

Cross-disciplinary innovations like technology, public administration, and citizen-centered design frameworks drive the development of smart public services. Their efficiency is just one of the numerous advantages these systems present. In fact, the promise of transformative change extends to the relationship between the government and citizens by documenting the shift toward more responsive, equitable, and effective public services.

### 3.4 Intelligent regulation and risk warning systems

Intelligent regulation and risk warning systems represent a significant advancement in government digital transformation, leveraging artificial intelligence to transform traditional regulatory approaches from reactive enforcement to predictive risk management. These systems integrate diverse data sources, advanced analytics, and machine learning algorithms to enhance regulatory effectiveness while optimizing resource allocation across various domains of governance.

#### 3.4.1 Conceptual framework and system architecture

Employs a multi-layered architectural framework for the optimisation of artificial intelligence-mediated governance in the governmental setting. It is accompanied by a diagram that illustrates the structured information flow across five interrelated functional levels, influenced by contextual factors comprising technologies as well as regulatory systems. The mutual feedback highlights learning adaptability which is critical for the effectiveness of the system.

The data acquisition layer merges disparate data sources such as IoT sensor networks, transaction data, regulatory documents, and external APIs. Such comprehensive integration permits regulated entities to surpass traditional reactive periodic inspections of oversight. Information from monitoring entities undergoes real-time processing, feature extraction, and integration in the processing layer to construct unified regulatory profiles, which go as a single entity.

Deviations from established regulatory thresholds are flagged by anomaly detection algorithms in AI systems at the analytical layer, while risk assessment models evaluate regulatory compliance risks and violation-preemptive forecasting models project potential breaches before they happen. Such approaches facilitate shifting the regulatory paradigm from enforcement-based reaction to proactive risk mitigation.

The risk warning system converts analysis results into early warning notifications, visualization aids for risks, and intervention alerts that are categorized by the magnitude of risk severity. The regulatory response system subsequently performs targeted inspections while enforcing compliance actions and adjusting regulations through trend identification and effectiveness evaluation.

**Figure 3:**
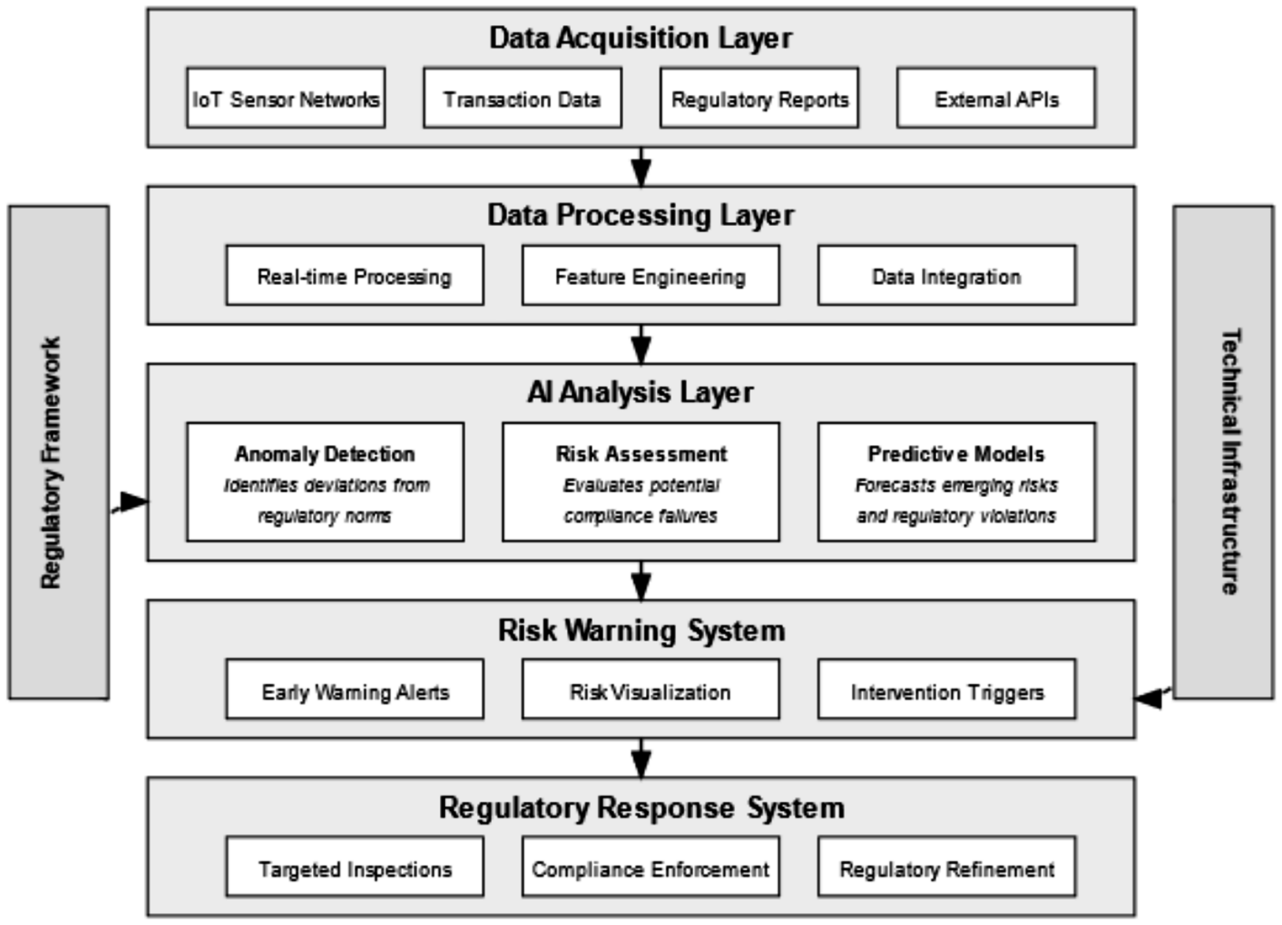
Conceptual framework of intelligent regulation and risk warning systems.

The feedback loops illustrated in Figure 3 highlight the learning capacity of the system, in that outcomes have associated feedback which, in turn, undergoes analysis for refinement. This circular mechanism enables the evolution of regulatory systems in relation to applied practices and patterns of compliance.

#### 3.4.2 Implementation cases and results

Intelligent systems of regulation have shown effectiveness in a variety of fields. For example, in environmental violation issues, sensor-based monitoring networks with predictive analytics have increased violation detection rates by 37 per cent while reducing the cost of inspections by 28 per cent. The financial sector has also benefited; the UK Financial Conduct Authority’s system was 42 per cent more accurate in early detection of market manipulation than the old system.

The framework for the design of public innovation governance should be based on a human rights protection system. It must address core issues such as control mechanisms and ethical responsibilities, and respond to deep-seated human concerns over excessive monitoring of control systems. In the legal framework of advanced artificial intelligence and algorithmic accountability, it is necessary to clarify compliance obligations, enhance system effectiveness, and at the same time simplify the effectiveness of control through risk-based precision.

## 4 Opportunities of AI-enabled government transformation

### 4.1 Enhancing decision-making scientific basis and administrative efficiency

Utilizing AI within various arms of the government presents unparalleled possibilities for augmenting the scientific basis of decision-making as well as the administrative efficiency of government bodies. Current AI technologies surpass anything previously achieved in their ability to process sophisticated data, detect relationships within data, and even form conclusions, which has the potential to shift systematized methods of governance.

Table 2 shows a comparison between traditional and AI-enhanced methods in five distinct areas of government decision making. The information provided in the table indicates that there have been improvements in the efficiency of processes and the quality of the results when AI technologies are used appropriately in public administration.

**Table 2:** Comparative analysis of AI applications for enhanced decision-making in government.

Application area	Traditional approach	AI-enhanced approach	Key technologies	Efficiency improvements	Decision quality improvements
Policy analysis	Manual review of limited data sources	Comprehensive analysis of multiple data streams	Machine learning, natural language processing	65% reduction in analysis time	42% increase in policy variable consideration
Resource allocation	Formula-based distribution with limited adjustment factors	Dynamic optimization with multiple contextual variables	Predictive analytics, optimization algorithms	38% reduction in resource waste	47% improvement in targeting precision
Risk management	Periodic risk assessments based on historical data	Continuous monitoring with real-time risk recalibration	Anomaly Detection, Predictive Modeling	53% faster risk identification	56% reduction in false positives
Regulatory compliance	Standardized enforcement schedules	Risk-based prioritization with adaptive monitoring	Machine learning, pattern recognition	41% reduction in compliance costs	36% increase in violation detection
Strategic planning	Expert-based forecasting with limited scenario analysis	Data-driven simulation with multiple scenario modeling	System dynamics, agent-based modeling	59% reduction in planning cycle time	44% improvement in forecast accuracy

The AI-aided policymaking rationale gets stronger on the empirical side with the ability of an AI to make concurrent purposive synthesis from multiple information sources. In policy analysis, AI systems can extract data from both structured and unstructured sources simultaneously which reveals relationships that, in standard analyzes, are very likely to go unnoticed. This advanced analysis permits the examination of policy constituents, context, and outcomes in a holistic manner.

AI assists in the optimisation of processes and also improves effectiveness in the refinement of administration. Resource allocation models based on static formulations are obsolete as dynamic algorithmic optimisation is now possible which continuously recalibrates allocation to ever-changing demands and performance benchmarks. Oversight compliance systems also benefit greatly from regulated resources governed by complex risk profiles instead of predetermined schedules under risk-based approaches.

With the advent of Artificial Intelligence, strategic planning can involve simulations that provide frameworks of complex histories made up of multiple interacting parts. AI also allows adaptive and contingency plans to be developed in contrast to forecasting which relies on expert intuition.

While these applications are promising, artificial intelligence decision-augmentation applications require careful attention to framed policies around data-digital security, algorithmic transparency, and the right balance of human participation. Effective systems tend to rely on practical norms and perimeter-based judgments regarding data collection, pattern recognition, capability development, and other achievable tasks; the decisions tend to be AI-based. This blend of humans and AI allows for ethical concerns of human decision-making to be fused with AI’s ability to use data to optimize processes and accelerate outcomes which, in countless novel ways, are grounded in scientifically proven frameworks.

### 4.2 Optimizing public service quality and precision

Integrating artificial intelligence into the public services offered by the government is an opportunity that can fundamentally change the method of delivery and improve the quality of services and the accuracy of government functions. By leveraging advanced data analytics, personalization algorithms, and predictive capabilities, AI technologies enable more responsive, customized, and efficient public services that better address diverse citizen needs.

Table 3 presents a comparative analysis of traditional versus AI-enhanced service models across five essential public service domains. The data demonstrates significant improvements in both service quality metrics and precision targeting when AI technologies are appropriately deployed in public service contexts.

**Table 3:** Comparative analysis of AI applications for public service enhancement.

Service domain	Traditional service model	AI-enhanced service model	Key technologies	Quality improvements	Precision enhancements
Healthcare services	Standardized treatment protocols with limited individualization	Personalized health interventions based on individual profiles	Predictive analytics, machine learning	37% increase in patient satisfaction	42% improvement in early intervention effectiveness
Social welfare	Categorical eligibility with standard benefit packages	Needs-based assessment with tailored support packages	Natural language processing, pattern recognition	45% increase in program effectiveness	53% reduction in service redundancy
Education	Standardized curriculum delivery with limited differentiation	Adaptive learning pathways with personalized content delivery	Intelligent tutoring systems, learning analytics	39% improvement in learning outcomes	48% better alignment with individual learning needs
Transportation	Fixed route and schedule systems	Dynamic routing with demand-responsive adjustments	IoT integration, predictive modeling	31% reduction in travel time	56% improvement in service availability
Administrative services	Process-oriented transactions with standardized procedures	Citizen-centric interfaces with anticipatory service delivery	Virtual assistants, automated processing	62% reduction in processing time	44% decrease in application errors

The quality improvement has achieved remarkable results in terms of citizen satisfaction, medical outcomes and the efficiency of services at all levels. The integrated medical system through the application of artificial intelligence data, with highly personalized diagnosis and treatment processes and proactive health management plans, significantly enhances patient satisfaction. For instance, the Danish National Health Service uses AI to analyze patients’ genetic data and medical history to formulate personalized treatment plans for cancer patients. As a result, patient satisfaction has risen from 60 to 82%. Similarly, integrated teaching projects that adopt artificial intelligence technology achieve a comprehensive improvement in teaching effectiveness by continuously optimizing personalized teaching resources, while also realizing precise matching and optimized upgrading of teaching content. For instance, in Finland, an intelligent education platform pushes customized courses based on students’ learning data. As a result, the pass rate of students in a certain region has risen from 70 to 97%.

Among social welfare service projects, the improvement in accuracy brought about by artificial intelligence technology is the most significant. By intelligently assessing demands and optimizing resource allocation, the problems of excessive or insufficient services that might occur in the traditional model have been replaced by a precise demand assessment system. This system can more precisely match service supply with individual demands, not only enhancing service efficiency but also optimizing cross-domain resource allocation. For instance, the social welfare department in Sweden has utilized AI to analyze the family structure, health conditions, and employment situations of applicants, and has formulated personalized welfare plans. As a result, the success rate of employment assistance programs has increased from 50 to 72%, and the rate of duplicate welfare distribution has dropped from 15 to 7%.

In the transportation sector, intelligent technologies have enhanced productivity. Demand-based automatic scheduling and dynamic routing further optimize service efficiency and availability. For instance, the public transportation system in Berlin, Germany, utilizes AI to analyze passenger flow data and road conditions in real time, dynamically adjusting bus routes and departure frequencies. As a result, the average travel time for a certain bus route has been reduced from 60 min to 41 min, and the punctuality rate has increased from 65 to 101%. In administrative services, productivity growth is most significant, thanks to the complex error-checking systems provided by virtual assistants and automated processing systems, which have improved processing time and accuracy. For instance, the AI passport application review system launched by the Australian Department of Home Affairs has shortened the review time from 7 days to 3 days and reduced the error rate of form filling from 20 to 11% through an automatic form review algorithm and intelligent customer service.

The application of artificial intelligence technology in service models has sparked important discussions on fairness, social equity and the efficiency of humanized services. Even in the face of complex social interaction scenarios, automated systems should at least achieve the following: when AI handles repetitive tasks, it should withdraw from intervention and instead provide proactive and personalized assistance around the clock. Only by meeting this condition can public services designed around specific individual needs achieve the best performance and ensure that the system operation reaches the optimal state. For instance, the AI system for older adults services in Netherlands automates repetitive tasks such as daily care appointments, while providing 24 h intelligent voice assistants for the older adults to answer health inquiries. This not only enhances service efficiency but also ensures humanized interaction.

### 4.3 Enhancing government transparency and public participation

The continuous growth of artificial intelligence (AI) technologies may provide new opportunities to improve government transparency, increase public participation, and effectively respond to informational accessibility concerns in relation to civic engagement. In regard to civic engagement, this has been an ongoing dilemma for some time now. Contemporary AI tools are providing higher-level access and mechanisms of information access which enable citizens to actively participate in public administration and policymaking processes.

Access to AI technologies that improve clarity and heighten civic participation at all levels of government enable citizens to engage and actively collaborate in the processes of policy formulation and decision making. Complex relationships within various domains of governance are presented visually and spatially as appealing dynamic images depicting critical policy decisions, budgetary expenditures, even administrative efficiencies as easy-to-grasp animated images citizens can readily access. Moreover, technical documents can be eloquently simplified by language processing tools, thus removing barriers in the information flow between the government and its citizens. Automated report generation functions provide powerful tools to effortlessly achieve real-time transparency in government information disclosures, thus enhancing the timeliness, accuracy, and completeness of information updates beyond scheduled manual updates.

The emergence of artificial intelligence technology has increased the value and effectiveness of interaction in a participatory sense. AI, for instance, effectively controls public discussion forums or what is known as digital deliberation, overcoming the limitations of face-to-face gatherings (on the order of thousands or millions of people). Through the use of AI tools, sentiment analysis and opinion mining, government organizations are able to gather and analyze public sentiment and perception about issues on a scale never before possible. Citizens’ attitudes that would normally be unearthed only by consultation methods that are hidden with conventional methods provide insights into attitudes concealed by traditional means. Tailored forms of participation allow for citizens to be matched with opportunities that align with their professional background and personal interests, thus enhancing both the reach and depth of participation.

Case providing implementations offer an Amsterdam example of administering these methods in practice. The “Open Algorithms” project allows citizens to access algorithmically executed local government decision-making processes with interpretive feedback interfaces. In South Korea, AI technologies are integrated into the “National Participation Platform” that scans countless proposals by citizen interest. AI determines consensus points and enables collaborative policy construction through formalized deliberation. In Finland, the “AI Assistant for Public Consultation” facilitates active retrieval of feedback on political documents making it possible for citizens to contribute textually, with the assistance during the consultations serving to increase input volume as well as participant engagement.

Taking the controversy over the “Transparent Budget AI Platform” in Mexico as an example, it reveals the risk of “digital exclusion” behind technological transparency—although the platform realizes the visualization of budget data, its interface only supports Spanish and requires at least 8Mbps of network bandwidth, resulting in 42% of grassroots people being unable to use it effectively. Based on this, this study proposes a “three-dimensional transparent evaluation system”: (1) Accessibility (language compatibility, device compatibility, network threshold); (2) Readability (information granularity, visualization complexity, proportion of professional terms); and (3) Interactivity (feedback response speed, opinion collection rate, diversity of participation channels). Taking Norway’s “Climate Policy AI Consultation System” as a positive case, this system has increased the public participation rate to 68% through “multilingual support + offline access function + voice interaction interface,” which is 257.9% higher than the traditional online questionnaire. At the same time, it has established a “suggestion adoption tracking mechanism,” publicly displaying the processing progress of each public suggestion, with an adoption rate of 31%. Significantly enhance public trust.

The unwarranted application of such recent frontier technologies undoubtedly holds risks. Implementing AI as a tool for encouraging participation and ensuring policy transparency requires careful deliberation. Designers of technological transparency should not succumb to too much reliance on algorithms that their workings, often characterized as black-box systems, hinder transparency themselves. Gaps concerning the accessibility of technology as well as requisite skills must be eliminated in order to provide for equitable participation across demographic divides. Technology’s role should be that of a facilitator of democratic discussions rather than a substitute. As such, equilibrium between authentic human involvement and AI must be determined.

Incorporating artificial intelligence in terms of engagement and transparency can improve the circulation of information, expand the range of engagement, and make policies, within a democratic scope, more attentive to citizens’ needs.

### 4.4 Promoting data sharing and departmental collaboration

The adoption of Artificial Intelligence technologies offers a unique set of advantages, particularly concerning ease of collaboration both within and between government agencies. Moreover, these technologies can aid in restructuring bureaucratic systems into more agile and integrative frameworks of governance. The AI technologies in use guarantee meaningful information exchange and cross-organizational collaboration in the resolution of complex, multi-dimensional problems which transcend defined organizational boundaries in an efficient and secure manner.

Enhanced information sharing stems from a number of technological options. Sophisticated data integration technologies have the capability of merging disparate sets of data residing in different organizational silos into singular cohesive data products without standardization processes. Model crafting collaboration permits the withholding of sensitive information fragments while mitigating privacy as well as security risks during data sharing. Enhanced privacy preserving data sharing increases the ease of sharing sensitive information among participants. Proactive intelligent information retrieval systems are capable of identifying and retrieving associated information from various departmental silos, resulting in reduced costs for access and retrieval.

Through the use of artificial intelligence, its benefits also extend to fostering inter-departmental cooperation through supporting joint actions and collaborative analysis. Multi-departmental consortia, even with gaps in their diverse disciplines and institutional frameworks, are able to form a common understanding of complicated problems through the use of AI Multi-Interpretative systems for Integrated Diagnostics and Reporting – an example of AI-assisted analytics systems. Complex, inter-organizational proprietary workflows can be managed by Coordination intelligent systems employing sophisticated job allocation techniques, ensuring proper order, as well as responsibility attribution despite the absence of centralized control mechanisms. Anticipatory multidisciplinary collaborative problem modeling strengthens the ability to pre-emptively deal with emerging problems before they materialize, instead of adapting plans after the fact reactive coordination executed post-problem emergence.

The exact matching of technical proficiency successfully addresses the inherent difficulties that have traditionally hindered coordination among governmental agencies. Gaining practical judgment accompanied by familiarity with important data sources lessens the information asymmetry within a given organizational structure. Completing collaborative work is greatly automated by processes like careful AI-powered discovery, aggregation, and coordination, which also lower the working costs. Data sharing barriers to inter-organizational collaboration and cultural silos entrenched within the organization could also be resolved by AI data sharing, quipped “operation enhancements”, of course with critical safety and privacy measures “cut in” as though glued.

Specific scope of challenges regarding implementation can be resolved by the adoption of appropriate governance frameworks. Legal governance policies must allow encrypted data sharing that’s responsible without gaps for careless blunders. Inter-division conflicts regarding the quality of information require governance procedures through managed validation and confidence processes. Organizational norms also require realignment bolstering proactive support aimed at collaboration instead of information control. Strategic investment toward agile infrastructure techniques also provides implementation for secure exchange standards of the system.

These expected advantages will be realized, which will further augment enhanced governance. Engagement adds coherence in policies as cross-system and interdepartmental fusion problems are dealt with more efficiently. Services are better integrated and become more responsive in relation to the demand from citizens, thus transcending administrative boundaries. There is broader use of composite information and collective reasoning for the analysis of social complexities from climate responsiveness to social vulnerability. If optimally leveraged, the information exchange enabled by AI technology, alongside interdepartmental collaboration, has immense transformational capacity to replace governance in departmental silos with integrated cross-departmental problem solving.

## 5 Ethical challenges in government AI applications

### 5.1 Algorithmic fairness and bias issues

Incorporating artificial intelligence technology into public sector functions profoundly complicates already-existing fairness and bias ethics frameworks, which along their social implications could be extremely detrimental to social equity and administrative justice. The application of AI by administrative bodies in core decisions that control citizens’ rights and dictate access to resources demands equity across demographic divides on ethical fairness thresholds that require serious deliberation.

**Figure 4:**
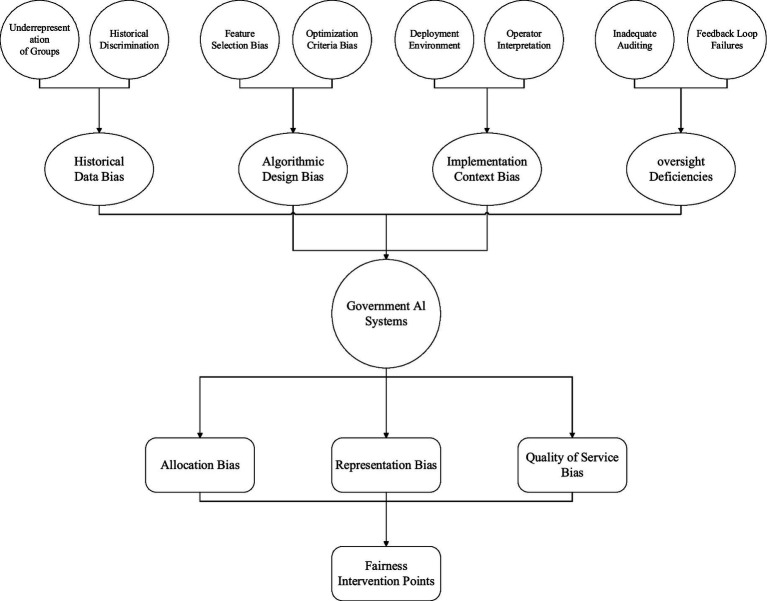
The origins and impacts of algorithmic bias in AI systems within government frameworks.

As Figure 4 shows, there are multiple types of algorithmic bias regarding the ways governments make use of it. It points out four fundamental origins of bias: data historical scope biases, algorithm design biases, contextual implementation biases, and supervisory control system dysfunctions. In the framework of government AI, these classifications come together to yield three broad ranges of outcome bias: outcome allocation bias, outcome representational bias, and service level outcome bias.

One particular aspect of concern within the scope of bias in historical data is that the AI systems built using historical government data cannot simply reproduce existing patterns of discrimination and underrepresentation. Algorithms trained on data representing outcomes or decisions made in the past, which incorporate biases present in those datasets, are capable of reproducing such biases in otherwise objective computational systems. This is most pronounced in areas such as the criminal justice system where discriminatory enforcement patterns are built into risk assessment tools and social welfare where longstanding patterns of resource distribution create the perception of institutional bias irrespective of actual need.

The choices that are made when constructing a model, from neutral decisions to population features, are the root causes for algorithmic design bias. Even when applying neutral and objective mathematical procedures, decisions such as determining which features to include, setting optimisation criteria, and establishing thresholds often with neutral and objective mathematical techniques highly polish a model’s fairness outcomes. Evidence has shown that quite a number of empirical attributes depend on equally neutral claims regarding the model structure, including protected attributes such as race, gender, and socio-economic status, which has direct impacts on fairness.

Contextual organizational structures of bias lead to gaps from a user’s explanation of the system’s output and the unequal chasm that exists between various societal groups where artificial intelligence systems are deployed. Besides an explanation of the output system, this type of bias underscores algorithmic output divergences in socio-technical gaps leading to non-neutral fairness concerns. In government institutions and organizations, sharp divides in perception concerning algorithmic decision support systems information, and recommendations arise as a result of the underlying decision-making culture and procedural logic shaping the system.

The absence of proper oversight in the form of faulty auditing and dysfunctional feedback loops provides an opportunity for the accrual of bias over time. Gaps in the supervision and responsibility mechanisms permit the unattended deterioration of government artificial intelligence infrastructure. This justification points out the need for continual maintenance as well as concern of bias rectification from the theoretical design phase.

### 5.2 Data privacy and protection of citizen rights

The adoption of artificial intelligence in government functions poses significant ethical concerns related to information privacy and the protection of citizens’ rights. The difficulties stem from the fact that AI systems require large amounts of personal data in order to provide satisfactory services, thus placing governments at the awkward intersection of advancing technology while preserving privacy rights and civil liberties.

Table 4 identified five underlying, critical challenge categories at the intersection of government AI systems, data privacy, and citizen rights. Public sector implementation of AI comes with several governance challenges, all of which stem from responsibility and ethics.

**Table 4:** Key data privacy and civil rights challenges in government AI systems.

Challenge category	Description	Ethical implications	Governance considerations
Data collection scope	Extent and methods of personal data acquisition by government agencies	Risk of surveillance overreach and chilling effects on civil liberties	Need for clear legal limitations and proportionality requirements
Purpose limitation	Adherence to specified purposes versus function creep in data usage	Potential for unauthorized secondary uses violating citizen expectations	Implementation of purpose specification frameworks and usage auditing
Consent mechanisms	Quality of consent obtained for data processing in asymmetric power relationships	Questions of voluntary and informed consent in mandatory government interactions	Development of alternative legitimacy frameworks beyond consent
Algorithm transparency	Visibility of data processing logic in government decision systems	Black-box decision-making undermining due process rights	Requirements for explainability in rights-impacting applications
Data security	Protection against unauthorized access and data breaches	Potential for identity theft, discrimination, and other harms	Implementation of security-by-design principles and breach protocols

Government use of AI comes with a great need to acquire personal data, which poses the fiercest threat in terms of privacy and individual freedom. The dichotomy stems from hyper-performance capability alongside ever-reaching surveillance potential, each threatening personal identity, freedom of association, and self-governance. Hence, strong data retention policies and stringent necessity regulations from these opposing principles are impactful in ensuring an equilibrium toward proportionality in data collection access.

Definable boundaries on data spending capabilities generate insignificant space filled with potentially numerous hazards and significant gaps created due to information collected by one government agency misallocated by other un-gated “intended” purposes. This scenario offers a high-def solution to the problem of function creep and undermines public expectation and confidence—these concerns heighten when viewed against a backdrop of unbalanced power dynamics between citizens and the state, amplified by the absence of consent frameworks which long ago became outdated.

The lack of understanding of the breaches includes privacy violations due to a lack of understanding of breaches and transparency of algorithms. Not revealing the application of algorithms in processes that lead to human rights abuses attacks the protective walls due process constructs for defenders. Similarly, uncontrolled access to government databases creates enormous residual risk harms because of the sensitive and classified nature of the information.

To remedy these concerns, governance frameworks tailored to these sorts of problems should strive to include efficient designs of boundary lines between law and technology within social systems where citizens and government institutions interact in democracies as complex adaptive systems.

### 5.3 Decision transparency and explainability deficits

The use of AI technologies in government functions poses major ethical challenges concerning accountability and transparency. As public administration and policymaking undergo transformation through the infusion of AI into higher functions and decision-making processes, there is a profound internal conflict between AI’s hidden systems of algorithms and the democratic principles of public value, public administration, and administrative justice.

Take the German “AI Welfare Qualification Review System” as an example. The system has raised public doubts due to its “black box” feature—83% of the rejected applicants said they “could not understand the reasons for rejection,” leading to a 300% increase in administrative lawsuits. To address this issue, this study introduces a “hierarchical interpretability framework”: (1) User level (for the public): Provide “natural language interpretation reports,” including “key influencing factors (such as income, family size) + decision-making rules (if income exceeds X yuan, it does not meet the requirements) + objection channels”; (2) Regulatory authorities (for administrative departments): Provide “model structure visualization,” showing the ranking of feature importance (calculated using SHAP values, such as an income feature SHAP value of 0.62, which is the most critical factor) and decision-making paths; and (3) Technical Layer (for developers): Provide “algorithm parameter details,” including model type, hyperparameter Settings, training data distribution, etc. After the framework was piloted in a certain state of Germany, the public understanding rate rose to 76% (compared with 21% before), and the number of administrative lawsuits decreased by 68%. At the same time, it passed the “consistency test of interpretation” (the logical deviation of interpretation in different cases was ≤5%) to ensure the reliability of interpretation. In addition, an “interpretation quality assessment index” was established: including completeness (coverage rate of key factors ≥90%), accuracy (consistency between interpretation and the actual decision-making logic of the model ≥95%), and comprehensibility (public cognitive load score ≤3 points, on a 1–5 point scale). All three indicators of the pilot system were met.

Unexplainability occurs in any phenomenon within three main limits concerning governance. The first limitation arises from the artificial intelligence technological decision-making processes based on sophisticated mathematical models like deep learning algorithms; even the creators cannot adequately explain the processes involved. Furthermore, such technological boundaries are worsened by conflicts between explanation and accuracy; the pursuit of models crafted to yield maximal explanations is often undertaken with meticulous computational techniques which, by their very nature, defy straightforward rationale.

Opacity arises due to a lack of adequate documentation alongside vague processes that obscure how algorithms are integrated into automated decision-making systems. Algorithms suggest decisions, but in certain circumstances, the individuals in charge of the final decision do not clearly explain what their reasoning is and why they choose to disregard the algorithms’ proposals. These kinds of systems tend to lack sufficient remedying obstacles to controllability silos devoid of responsible attribution systems to shield automated and human action where rational and empirical blame and causal determination can be elucidated.

They are compounded by the lack of defined institutional boundaries as government entities use proprietary restrictions, security precautions, and advancements in technology aimed at reducing external focus on algorithmic processes to conceal scrutiny deterrence. In many jurisdictions, there is a lack of openly accessible information sought by the general populace, especially regarding the existence and use of algorithms, what regulates such algorithms, how they are evaluated, or operational benchmarks—especially for systems dealing with vulnerable populations.

These explainability gaps have considerable ethical consequences on the democratic governance structure. From the perspective of procedural justice, impacted individuals lack the meaningful ability to contest decisions that, at their core, are beyond their comprehension, which violates due process, particularly when algorithms make decisions affecting an individual’s freedom in criminal justice, immigration, and welfare adjudication interfaces. Trust in governmental institutions is also damaged when citizens view automated systems as opaque and beyond control.

To influence all these aspects simultaneously requires both legal and institutional action which is multi-faceted and comprehensive. From the perspective of technology, this means creating explainable model architectures where appropriate, utilizing post-hoc explanation techniques such as LIME or SHAP for intricate models, employing counterfactual reasoning to provide valuable relevance tailored to varying degrees of impact. Legal “right to explanation” is increasingly appearing in regulations, but the dividers of contextual accessibility, level, structure, and explanation adequacy remain detrimental for defined groups.

More fundamentally, government agencies need to conduct context-specific evaluations on balancing the complexity and interpretability of models based on the decision context and the stakes involved. In approach prioritization for explainability, in some low-accuracy models, a modest performance trade-off can be made in order to ensure interpretable decisions for the primary stakeholders, thus enabling democratic governance and administrative legitimacy within a democracy.

### 5.4 Blurred responsibility boundaries and accountability mechanisms

The adoption of artificial intelligence technologies within the structures of government poses a profound threat to conventional forms of accountability. Blurred and indistinct boundaries of responsibility create a multi-level supervisory and control action deficit. This lack of balance is worsened by a combination of poorly designed organizational systems, overly structured legal frameworks, and an evolving blend of governance algorithms, all of which are resistant to change.

A fundamental concern regarding accountability for artificial intelligence systems stems from their socio-technical character, allocating responsibility to disparate human and non-human actors. Each of these types of actors has a role in the provision of data, supervision of function-performance within particular institutions and operational execution of algorithmically dictated procedures. In this case, negative results cannot be sufficiently linked to any singular actor within the decision-making processes literature ascribed to “responsibility gaps.”

The technical aspects of the AI system restrict its external auditing, thus creating cross-organizational blame for the system as a whole. Another feature of sophisticated AI systems is non-deterministic behavior, where systems provide different results from the same input. This makes it extremely difficult to define system-wide standards of care or performance metrics. Furthermore, the absence of definitive human oversight gives rise to temporal problems relating to responsibility. There are voids of decision-making where autonomous action drifts beyond the prescribed boundaries without human oversight due to the evolving capabilities of machine learning systems.

These structural divisions are further exacerbated by the character of these organizations which result from diffused governance arrangements. An AI system within the government is comprised of multiple organizational units which include external contractors, policy units, operational units, and information technology units. Fragmented workflows create additional coordination barriers and diffusing levels of accountability result in coordination problems. In addition to the spiral of fragmentation, procurement policies purposely directed toward technical efficacy drastically reduce transparency and accountability, thus serving to further compound structural weakness.

The responsibility of human law is under considerable stress due to the challenges posed by algorithmic decision-making. An aspect of administrative law typically operates within the bounded assumption that human agents, one at a time, are in control of decisions made within predefined parameters and thus are accountable for those decisions. Unlike AI systems, where the technological and human components of a system share the decision-making processes and hence blur the boundaries of responsibility, artificial intelligence systems allocate decision-making responsibilities to both human and technological components making it difficult to apportion responsibility. The same issues arise in tort law where causation and breach of duty in complex algorithms, which are dependent on a blend of code, data, and the context in which it is deployed from the level of which the outcome is determined, are all difficult to disentangle.

Responsibility centered around these forms of accountability needs to be solved through more comprehensive solutions which reframe the boundaries of algorithmic governance. The construction of governance around algorithmic systems should focus on laying effective mechanisms to control systems before a set date. These should clearly delineate standards, control processes such as algorithmic impact assessments ahead of time, and outline multi-stage testing requirements, definition of expectations among other performance indicators. Simultaneously, systems should permit continuous monitoring through audit trails, validation, operational control by expert governance non-executive directors in addition to significant controller powers. Channels to appeal to decision-making processes in post-operational measures, review systems on a set timetable alongside liability for system failures are also vital.

The use of artificial intelligence by governing bodies surely demands a reshaping of the concept of responsibility which combines both the collective aspect and the need for unambiguous definitions on various levels. This approach accounts for the socio-technical interactions related to the creation of an algorithm, as for example, well-defined institutional organizational policies, professional norms legislated in laws, and commensurate sanctions for breach of such norms. Without such comprehensive frameworks of accountability, the democratic legitimacy of regulation by algorithms is seriously eroded.

### 5.5 Digital divide and social inclusivity

The use of artificial intelligence technologies by the government raises important issues of digital inequalities and social inclusion. As government services adopt automated systems powered by AI technologies, the entrenched socioeconomic inequalities will likely be exacerbated by the unequal distribution and access to these technologies. This kind of technological stratification goes beyond barriers to access and includes gaps in social competencies, availability of relevant devices, and algorithmic representation bias.

Those at the bottom of the social hierarchy face additional barriers to accessing government services, especially when AI technologies are utilized. In this case, the examples are the older adults, language minorities, persons with disabilities, and economically disadvantaged. Exacerbated restrictions to the access of services, administrative burden, and erosion of entitlement to services due to the implementation of complex technological systems offers one clear illustration. In this case, a curious paradox arises: those individuals who most need government services are, paradoxically, the ones least able to access them in their new, digitally mediated versions.

Beyond individual access, ethical issues impact deeper on a democratic division regarding representation. When whole segments of society are subject to systematically imposed barriers to engaging with AI-driven government services, the validity of the representativeness of input data and recurring feedback loops is fundamentally compromised. This creates a situation of negative feedback loops whereby AI systems are perpetually recalibrated to serve the needs and interests of already dominant and, thus, marginalizing these digitally disadvantaged groups in access terms.

These issues require comprehensive strategies which improve access for all, provide various means of entry to a system, accommodate different tiers of digital literacy, and emphasize community-based gatekeepers. Above all, any new policies on the use of artificial intelligence technologies should center social inclusion as the foremost priority in the design processes. This remains important because social equity concerning technology must integrate into the administrative justice paradigm and governance upholds the democratic legitimacy of contemporary politics.

## 6 Construction of ethical governance framework for government AI

### 6.1 Ethical principles and value orientations

The application of artificial intelligence technologies in governmental functions must tackle emerging issues around algorithmic governance to ensure that such applications adhere to the principles of democracy. In addition to the ethical considerations set forth in Section 5 and the theoretical framework discussed in Section 2, this, too, would satisfy the requirements of flexibility merited by the technological pace in the world today in a way that is devoid of a normative straitjacket.

Artificial intelligence ethics in the governance domain must position humanity as the primary concern for all public services as the axis of restriction where welfare, dignity, and autonomy dominate far supersede efficiency in administration or technological streamlining. In relation to people, these principles must ameliorate the entrenched inequalities in government-citizen relations exacerbated by algorithmic governance which pervasively designed systems can fortify, slope, or mitigate entrenched discrimination due to their systemic design and intended applications. In the context of a democracy, administrative regulations on algorithmic governance must accentuate algorithmic oversight.

**Figure 5:**
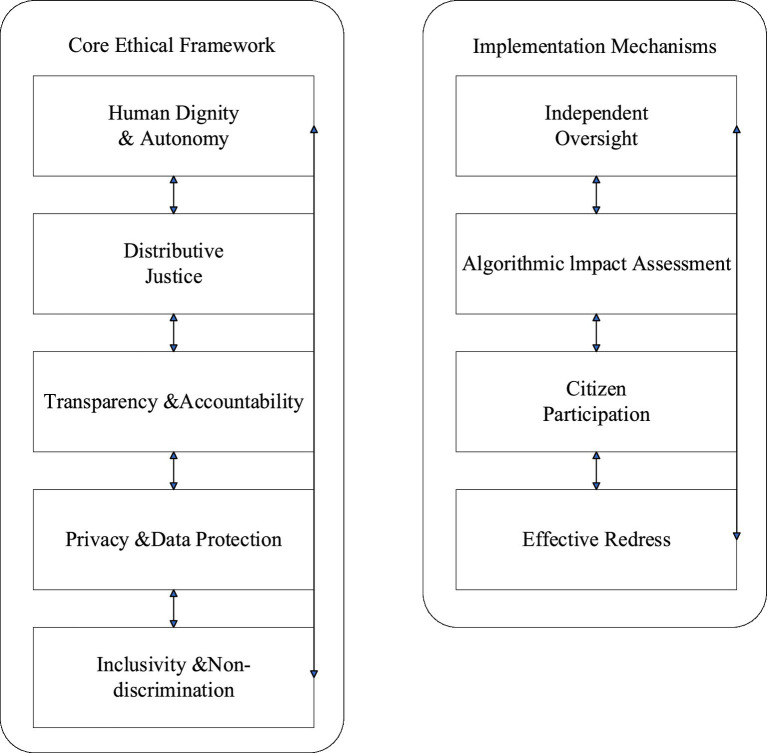
Ethical governance framework for government AI applications.

Figure 5 illustrates the central relationship of the basic ethical principles and the techniques required to manage artificial intelligence within the context of public administration. The inner circle illustrates the value system that should guide the development and functioning of the AI system whereas the outer circle illustrates the operational system which embodies these principles in practice. The interrelated two-way relationships are explained by the use of bidirectional arrows suggesting that a reasonable ethical governance of AI in public administration requires synergy rather than an isolated approach.

### 6.2 Ethical design technical solutions

#### 6.2.1 Explainable artificial intelligence

Explainable AI (XAI) resolves the transparency gaps identified by offering technical means to explain the operation of algorithmic systems used in government contexts. As algorithmic systems increasingly automate administrative processes that have far-reaching societal implications, the ability to provide human-understandable explanations becomes critical for the maintenance of democratic accountability and procedural justice.

For complex neural network models deployed in government applications, Local Interpretable Model-agnostic Explanations (LIME) provides a formal approach to approximating local behavior of the model f around a specific prediction x using an interpretable model g . This can be formulated as Equation 1:


(1) $$\min_{g\in G}L(f,g,\pi_{x})+\Omega (g)$$

Where ℒ represents the locality-aware loss function measuring how well the interpretable model g approximates the original model f in the neighborhood of instance x defined by $\pi_{x}$ , while Ω(g) represents the complexity of the explanation model. This approach allows the authorities to gain insight into the importance of particular features for specific decisions, especially useful in critical areas such as assessing eligibility for benefits.

#### 6.2.2 Privacy-preserving computation

The methods of privacy preservation in computation offer the described opportunities for data utilization while mitigating the challenges around privacy protection. The approaches also enable government agencies to obtain and analyze intelligence without infringing on the privacy of individuals, thereby preserving essential public trust in the government.

In the scope of government data analytics, differential privacy offers the basic principles in the form of a mathematical framework for measuring and bounding privacy leakage simultaneously. A randomized mechanism ℳ satisfies ϵ -differential privacy if for all datasets $D_{1}$ and $D_{2}$ differing on a single element, and all subsets S of the range of ℳ :


(2) $$P[M(D_{1})\in S]\leq e^{\epsilon }\times P[M(D_{2})\in S]$$

Privacy, as formally defined through the Equation 2, means the absence or presence of any individual record will not bring about any meaningful influence to the outcome of any analysis. This property is particularly beneficial when publishing government statistics or performing predictive modeling on sensitive demographic data while still retaining robust privacy guarantees.

#### 6.2.3 Fair algorithm design

Worry about biases within algorithms can be solved with appropriate algorithm design strategies which enable the implementation of fair AI systems across various segments of the population. These methods incorporate fairness into the construction of the algorithm rather than treating it as an afterthought.

One example of formalized algorithmic fairness that can be integrated into public AI systems is the statistical parity difference. For a classifier h , sensitive attribute A (e.g., race, gender), and outcome Y , this metric can be expressed as Equation 3:


(3) $$\Delta_{\mathrm{SP}}(h)=|P(h(X)=1|A=0)-P(h(X)=1|A=1)|$$

Where a smaller $\Delta_{\mathrm{SP}}$ indicates more equitable predictions across demographic groups. Alternative fairness metrics include equalized odds, which requires equivalent false positive and negative rates across groups, formalized as Equation 4:


(4) $$P(h(X)=1|A=0,\;Y=y)=P(\begin{array}{l} h(X)=1|\\ A=1,\;Y=y \end{array})\;\text{for}\;y\in 0,\;1$$

The selection of fairness metrics relevant for consideration in government AI systems requires contextual understanding since various definitions of fairness might be suitable for different administrative use cases, in addition to the fact that social equity in public governance should be observed at a higher level when forming the mathematical definitions.

### 6.3 Legal regulations and policy safeguards

The policies and legislative frameworks governing the proposed socio-technical approaches will aid in enhancing the practical implementation of AI explainability and equity algorithms within the government. The practical boundaries provided by governance theories suggest that the harsh boundaries of AI law should enable its public sector administrative multifaceted application. There must be specific criteria with regard to the assessment of algorithmic impact, strong enforcement review bodies, and comprehensive allocation of responsibilities for damages due to AI in state activities.

The protective policy proposals with multi-tier governance structures must incorporate graded supervision proportionate to evaluated risk levels whereby the most stringent criteria are applied to measures that pose high threats to fundamental rights or critical resource allocations. These structures derived from polycentric governance theories along with other scholarship would allow local ethics review bodies to national specialized technical entities and other constituencies to participate in centralized complete oversight. Moreover, contracting processes offer opportunities to advance the agile ethical AI agenda by mandating nonsubstantive business process transparency, fairness, and privacy guarantees before engagement with public administration systems.

### 6.4 Multi-stakeholder collaborative governance mechanisms

Accompanying the increasing difficulties concerning the regulation and governance of AI, socio-political ethical issues will need to be addressed by multi-faceted governance approaches. Formulated ethical AI frameworks, as articulated within collaborative governance theory, require synergistic participation from government agencies, technology developers, civil society, and active stakeholders in order to transcend rhetoric by designing context-appropriate ethical frames and implementing strategies (15).

These multi-stakeholder governance frameworks face other elements of complexity such as formal consultations for each stage of the AI life cycle, from design to implementation and post-deployment evaluation, with different categories of experts. These arrangements are formalized under organizational bodies such as:

AI Ethics Councils which blend governmental, technical, and civil society stakeholders able to review powerful systems examined. Community Oversight Boards which empower local participants subject to local systems to actively engage to influence decisions around local concerns. Technical Standards Bodies which are devising adaptable ethical guidelines to be applied *in situ*.

Interdisciplinary Research Collaborations focusing on the governance aspects of AI and expanding scholarship on its societal impacts.

The cooperative models of governance analyzed undertook the refinement of consolidated oversights traced through vague boundaries of accountability by specific division of oversight functions with complementary safeguards aimed at preventing system breakdown. Also, these cooperative structures close the gap of the digital divide because they consider the views of communities that are peripheral to the government system. Their efficiency is conditioned by the right institutional framework that facilitates equity inclusiveness in critical participation through structured solutions to controversial interests among various divided actors based on democratic value principles.

## 7 Conclusion

The level and range of challenges posed by AI technologies to the process of the government’s digitalisation, including transformative shifts and the consideration of fundamental concerns in ethics, AI technology, and its comprehensive impacts.

There are several approaches to the global framework on artificial intelligence offered by several primary jurisdictions (16). These include the business market model, which aims to provoke innovation within industry, the state-led model, which underscores the need for national collective action, and a norm-generating system which emphasizes the establishment of ethical standards. Regardless of these distinctions, there is a growing recognition that innovation will provide the most effective solutions to the governance of AI only if proper boundaries are defined and ethical constraints are addressed.

As far as government administration is concerned, artificial intelligence can transform an unprecedented number of issues (17). The effectiveness and efficiency of administrative tasks have been significantly enhanced by the presence of intelligent decision support systems that improve the information backbone of administrative decisions. AI-enabled public services guarantee enhanced delivery of services to the citizenry through predictive and personalized AI systems. Citizens are aided by advanced data visualization and digital deliberation tools to promote participation while transparency in governance helps sustain trust.

AI-integrated data fusion technologies, as well as federated learning algorithms, catalyze the fragmentation of departmental data silos.

Such applications engage the ethics dimension, which requires advanced effort and deep consideration. Social discrimination is threatened on the scale of algorithmic discrimination because biased patterns that exist in the data are reaching the point where automated decision-making is taking place. The users’ privacy issues alongside information collection are especially problematic owing to the massive demand for personal information required for the optimal functioning of the AI systems, which causes citizens’ conflicting dilemmas. In many cases where advanced algorithms are implemented, far-reaching decisions are made in processes that lack transparency in resolution, leading to undermined visibility in the transparency balance while eroding crucial aspects of accountability in governance. Furthermore, within socio-technical systems the attribution of responsibility appears to be very unclear, particularly in the context of anticipating negative consequences where it is difficult to establish who is at fault.

Addressing such problems effectively requires the formulation of an all-encompassing governance framework comprising policy solutions, as well as technological, legal, and oversight approaches that cut across multiple actors. At a technical level, the principles of explainability in artificial intelligence are supported by the enforcement of privacy and fairness algorithms at the discretion of the algorithm’s developer. From the legal perspective, enabling responsible innovation is bounded by risk-sensitive regulation regimes and procurement requirements. A range of institutional arrangements defines accountability for oversight for diverse stakeholders, standard-setting and evaluation processes.

This study contributes to the theoretical model and practical scope of artificial intelligence in as far as public administration is concerned. It broadens the debate on the alignment of ethical technologies with reactive governance, thus broadening the framework for analyzing the public interest of AI. Also, it directs attention to decision-makers, administrative executives and technology experts seeking to optimize the opportunities presented by AI while instituting robust ethical safeguards. The impact of adoption on implementation in different government contexts should be examined in subsequent studies to understand the determinants of artificial intelligence (AI) adoption and its consequences. Parsing the governance frameworks may illuminate pathways to aid the responsible deployment of AI in governance. Answering ethical dilemmas of public sector organizations requires custom-built approaches to explainability, fairness, and privacy—especially in regard to government use—focusing on the mechanisms themselves (18, 19).

As AI drives a global push toward digitisation, there is a critical need for policies that advance social objectives while balancing ethical concerns that protect democratic ideals and public confidence. This study seeks to pave the way for harmonizing technological innovations with civic values in digital governance.

## Data Availability

The original contributions presented in the study are included in the article/supplementary material, further inquiries can be directed to the corresponding author.

## References

[1] Engstrom DF Ho DE Sharkey CM Cuéllar M-F. Government by algorithm: artificial intelligence in federal administrative agencies. Admin Conf U S. (2020) 36:1–123.

[2] Cath C. Governing artificial intelligence: ethical, legal and technical opportunities and challenges. Philos Trans R Soc A Math Phys Eng Sci. (2018) 376:20180080. doi: 10.1098/rsta.2018.0080, PMID: 30322996 PMC6191666

[3] Ahn MJ Chen YC. Digital transformation toward AI-augmented public administration: the perception of government employees and the willingness to use AI in government. Gov Inf Q. (2022) 39:101664. doi: 10.1016/j.giq.2021.101664

[4] Chatterjee S. AI strategy of India: policy framework, adoption challenges and actions for government. Transform Gov People Process Policy. (2020) 14:757–75. doi: 10.1108/TG-05-2019-0031

[5] Batool A Zowghi D Bano M. AI governance: a systematic literature review. AI Ethics. (2023) 3:423–38. doi: 10.1007/s43681-024-00653-w

[6] Mergel I Edelmann N Haug N. Defining digital transformation: results from expert interviews. Gov Inf Q. (2019) 36:101385. doi: 10.1016/j.giq.2019.06.002

[7] Janowski T. Digital government evolution: from transformation to contextualization. Gov Inf Q. (2015) 32:221–36. doi: 10.1016/j.giq.2015.07.001

[8] Gil-Garcia JR Dawes SS Pardo TA. Digital government and public management research: finding the crossroads. Public Manag Rev. (2018) 20:633–46. doi: 10.1080/14719037.2017.1327181

[9] Fountain J. The wicked nature of digital transformation: A policy perspective. Dubai Policy Review, (2019) 1:40–45. doi: 10.46993/DPR/EN005

[10] Lember V Brandsen T Tõnurist P. The potential impacts of digital technologies on co-production and co-creation. Public Manag Rev. (2019) 21:1665–86. doi: 10.1080/14719037.2019.1619807

[11] Wirtz BW Weyerer JC Sturm BJ. The dark sides of artificial intelligence: an integrated AI governance framework for public administration. Int J Public Adm. (2020) 43:818–29. doi: 10.1080/01900692.2020.1749851

[12] Roberts H Cowls J Morley J Taddeo M Wang V Floridi L. The Chinese approach to artificial intelligence: an analysis of policy, ethics, and regulation. AI & Soc. (2021) 36:59–77. doi: 10.1007/s00146-020-00992-2

[13] Taeihagh A. Governance of artificial intelligence. Polic Soc. (2021) 40:137–57. doi: 10.1080/14494035.2021.1928377

[14] Dwivedi YK Hughes L Ismagilova E Aarts G Coombs C Crick T. Artificial intelligence (AI): multidisciplinary perspectives on emerging challenges, opportunities, and agenda for research, practice and policy. Int J Inf Manag. (2021) 57:101994. doi: 10.1016/j.ijinfomgt.2019.08.002

[15] Wirtz BW Weyerer JC Kehl I. Governance of artificial intelligence: a risk and guideline-based integrative framework. Gov Inf Q. (2022) 39:101685. doi: 10.1016/j.giq.2022.101685, PMID: 41133208

[16] Butcher J Beridze I. What is the state of artificial intelligence governance globally? RUSI J. (2019) 164:88–96. doi: 10.1080/03071847.2019.1694260

[17] Larsson S. On the governance of artificial intelligence through ethics guidelines. Asian J Law Soc. (2020) 7:437–51. doi: 10.1017/als.2020.19

[18] Aoki N. An experimental study of public trust in AI chatbots in the public sector. Gov Inf Q. (2020) 37:101490. doi: 10.1016/j.giq.2020.101490

[19] Sun TQ Medaglia R. Mapping the challenges of artificial intelligence in the public sector: evidence from public healthcare. Gov Inf Q. (2019) 36:368–83. doi: 10.1016/j.giq.2018.09.008

